# Land-Use Effect on Olive Groves Pest *Prays oleae* and on Its Potential Biocontrol Agent *Chrysoperla carnea*

**DOI:** 10.3390/insects12010046

**Published:** 2021-01-08

**Authors:** João Frederico Alves, Sara Mendes, António Alves da Silva, José Paulo Sousa, Daniel Paredes

**Affiliations:** Centre for Functional Ecology, Department of Life Sciences, University of Coimbra, Calçada Martim de Freitas, 3000-456 Coimbra, Portugal; joaofredma@gmail.com (J.F.A.); sara.mendes@gmail.com (S.M.); antonioalvesdasilva@gmail.com (A.A.d.S.); jps@zoo.uc.pt (J.P.S.)

**Keywords:** biodiversity, conservation biological control, geospatial analysis, landscape

## Abstract

**Simple Summary:**

To rethink the counterproductive effects of the recurrent use of pesticides to control pests, we examine how a conservation biological control approach can promote the necessary conditions for the development of a natural enemy (*Chrysoperla carnea*) that controls olive moth pest (*Prays oleae*) in 25 olive groves of the Portuguese Beira Interior region. Our study has the distinctive peculiarity of joining varied technical approaches, since the databases contained information related to the abundance records of both insect populations, the record of olive fruits infestation by the pest, and the information obtained after a geospatial analysis that resulted in landscape metrics. Overall, we corroborated the attraction of *C. carnea* to the olive moth, highlighted the possible biocontrol potential of *C. carnea* on this pest, asserted that the promotion of the diversity of land-uses has a significant effect in reducing the abundance of pest, and confirmed that landscapes dominated by olive groves promote the development of *P. oleae*. The implication of these results is of extreme importance for olive growers since promoting land-uses complexity and heterogeneity surrounding olive groves can reduce the likelihood of suffering pest outbreaks and help to avoid associated economic and environmental problems.

**Abstract:**

Olive growing has been intensified through the simplification of agricultural landscapes. In order to rethink the environmental drawbacks of these practices, conservation biological control techniques have been examined. In this work, *Prays oleae* and its natural enemy *Chrysoperla carnea* were monitored to account for the effects of the amount and diversity of different land-uses. We found that *C. carnea* showed an attraction to areas with high abundances of *P. oleae* but this predator did not display any affection by the different land-uses. Inversely, *P. oleae* abundance was lower in diverse landscapes and higher in simplified ones. Importantly, higher abundances of *C. carnea* were related to lower infestation levels of *P. oleae* in the late part of the season. These results corroborate the attraction of *C. carnea* to the olive moth, highlighting the potential of *C. carnea* as a biological control agent of this pest, assert that the promotion of land-use diversity can reduce *P. oleae* and confirm that landscapes dominated by olive groves can promote this pest. The present study aims at contributing to the discussion about the management of agricultural ecosystems by providing farmers with sustainable alternatives that do not have harmful effects on the environment and public health.

## 1. Introduction

According to the United Nations (2015) [1], agriculture is the largest employer in the world, providing livelihoods for 40% of the global population. Since the last century, when there was a considerable increase in world population, powerful market pressures led to massive production in agricultural systems by converting diverse natural and semi-natural habitats into monocultures. In most of these mass-production agricultural systems, pest control is quite aggressive and relies on the use of agrochemical inputs such as pesticides. As a result of these practices there is a degradation of natural resources and contamination of fundamental systems of the biosphere such as air, soil or water, seriously jeopardizing public health [2,3]. The most obvious way to manage agricultural systems in a more resilient and sustainable way is to eliminate such external inputs by replacing the use of pesticides for strategies based on natural predator-prey dynamics such as conservation biological pest control.

Conservation biological pest control is a strategy based on the use of natural occurring organisms to suppress population density or the impact of a specific pest organism, making it less abundant than what they would be if these organisms were not used [4]. It does not require continuous input of mass-reared natural enemies and avoids problems that may be caused by introducing exotic organisms into the environment by other biocontrol approaches [5,6]. It is based on the manipulation of the environment, at local (e.g., ground cover, hedgerows) and at landscape scale (e.g., proportion of natural habitat in the surrounding area), to enhance the survival, fecundity, longevity, and behavior of natural enemies already present in the agricultural ecosystem [7,8]. Those practices require the creation of a sustainable environment to balance the relationship between crop, pests and natural enemies in order to avoid production losses [9]. Despite being based on natural interactions, it often has a direct human influence and is widely used in integrated pest management (IPM) programs [10] along with other control strategies.

Despite the success shown by local conservation biological control techniques [11,12], ecologists, agronomists, and farmers are increasingly recognizing the critical role that surrounding landscape can play in determining pest damage [13]. Simple and homogeneous landscapes are generally originated from mass-production agricultural systems, defined by continuously extended agricultural fields and by reduced diversity of vegetation [14]. This type of landscape limits the availability of refuge and resources that non-crop habitats offer to pests’ natural enemies, emphasizing pest pressure over the farming systems [15,16]. On the other hand, complex landscapes covered by natural or semi-natural habitats, such as diverse woodlands, grasslands or shrublands are important to favor the prospection of natural enemies by providing undisturbed areas that offer shelter from crop disturbances as well as overwintering refuges, alternative hosts and prey, and additional nectar resources [7,17,18,19]. Consequently, the diversity and abundance of available natural enemies to provide biological control in agricultural systems also depends on the composition and structure of the surrounding landscape [7,8,20,21]. Two complementary mechanisms are thought to underlie landscape effects on pests and their natural enemies [22]. First, the resource concentration hypothesis states that expansive monocultures allow specialist pest populations to rapidly build and disperse [14,23,24]. Second, the natural enemy hypothesis recognizes that many natural enemies of crop pests (i.e., predators and parasitoids) depend on a diversity of crops and/or natural habitats for alternate food resources, overwintering, etc. [8].

Olive groves are affected by the olive moth, *Prays oleae* (Bernard) which is an insect belonging to the order Leptidoptera. It feeds exclusively on olive trees, and therefore it is monophagous and one of the major pests in Southern Europe olive groves, together with the olive fly, *Bactrocera oleae* [25,26,27]. It has three different generations that subsequently feed on the leaves (phyllophagous generation), the flowers (anthophagous generation) and the olive fruits (carpophagous generation). The damage caused by this lepidopteran becomes more evident in the years of low harvest [28]. The destruction of leaves, flowers, and the early fall of fruits caused by this pest can compromise the annual production and even the development of olive trees in the following years [25,27,29,30].

*Chrysoperla carnea* larvae are major oophagous predators known to play a predominant role in the predation of eggs of *P. oleae* [10,31,32,33]. In contrast to larvae, *C. carnea* adults are not predaceous and mainly feed on substances of vegetal origin such as nectar, pollen, and honeydew [34,35,36], adults of these insects are highly mobile organisms which are strongly affected by landscape composition because their biology, behavior, and dynamics depend on spatial distribution of resources [37,38]. For example, the pollen produced by groundcover flower strips favored its reproductive process [11,12]. Other studies suggest that the proportion of semi-natural habitats increase both abundance and diversity of adults and eggs of lacewings in vineyard landscapes, although that effect varies over time [39]. Additionally, vegetation diversity promotes a higher abundance of larvae on olive groves during the olive moth egg-laying period, indicating a great potential for conservation biological control approaches [40].

Here, we study the potential effectiveness of *C. carnea*’s biological control on *P. oleae* in olive groves, identifying the dynamics of their relationship in relation to different land-uses and with landscape diversity. We specifically aimed to answer the following questions: (i) Do different types of land-uses in the surroundings of the olive groves and landscape diversity have an effect on *C. carnea* and *P. oleae* abundance? (ii) Does *P. oleae* have an effect on *C. carnea* populations? (iii) Is there any effect of *C. carnea* on the infestation of the carpophagous generation of *P. oleae*?

## 2. Materials and Methods

### 2.1. Study Sites and Landscape Analyses

**Figure 1:**
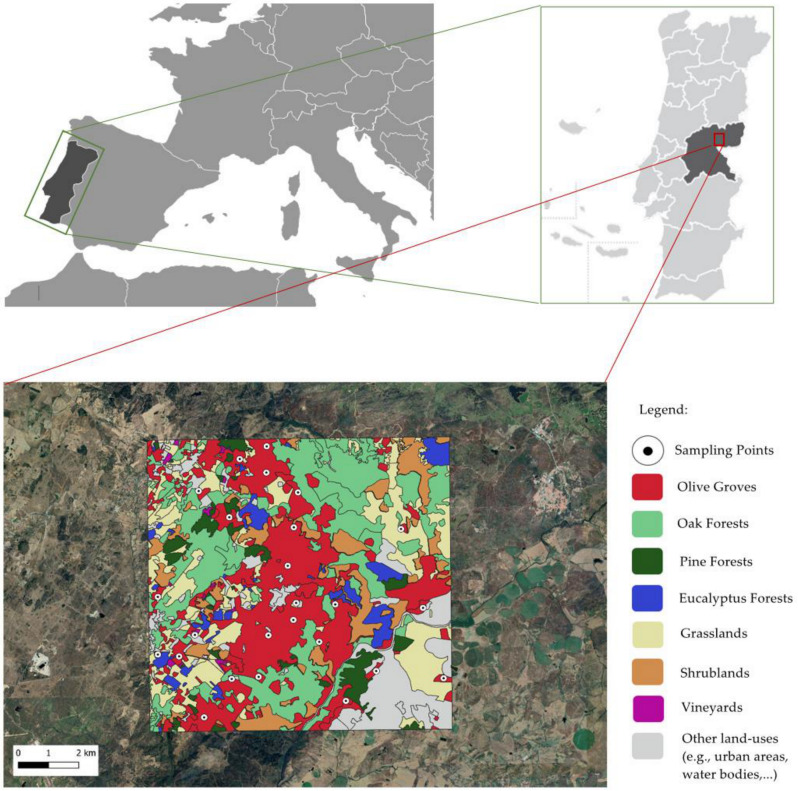
Illustrative representation of the exact location of the twenty-five sampled olive groves.

To accomplish the objective of this study, twenty-five olive groves were selected within the Beira Interior region of Portugal within the municipalities of Castelo Branco and Idanha-a-Nova (Figure 1). Olive groves selection criteria tried to keep the sampling points separated by a distance of at least one kilometer, in order to maintain spatial independence. However, this minimum distance did not always reach one kilometer, due to physical impossibilities related with the natural layout of the olive groves properties. The minimum distance between points was 588.1 m, while the maximum distance was 10,331.8 m (Figure 1). Sampling point selection criteria also had to allow them to follow a gradient of landscape complexity measured with the Shannon diversity index. The twenty-five olive groves where the sampling points were located were mostly centenary, non-irrigated and with low groundcover vegetation due to livestock presence. During this study, agricultural managers did not apply pesticides and did not use land ploughing methods.

At each one of the 25 sampling points, a geospatial analysis of the surrounding buffer area with a radius of 500 m was done by using the QGIS software (Open Source Geospatial Foundation, Beaverton, OR, USA), a Geographic Information Systems (GIS) platform. Based on satellite pictures, we generated polygons by depicting the different patches of the different land-uses found in the study area. These were: olive groves, oak forests, pine forests, eucalyptus plantations, grasslands, shrublands and vineyards. To validate this delineation of the landscape elements, as well as adding data to the elements that cannot be identified in the aerial photographs, it was necessary to verify the existing vegetation during the periods in the field. All this geospatial information was converted into raster images and it was inserted in the Fragstats software (University of Massachusetts, Amherst, MA, USA). From this spatial pattern analysis program, we obtained landscape metrics at the class level, in which the total area of each patch within each of the landscape buffers was quantified through the percentage of landscape (PLAND) and at the landscape level in which Shannon’s diversity index (SHDI) values were quantified [41].

### 2.2. Insect Sampling

In order to determine the abundance of the populations of the pest *P. oleae* and the predator *C. carnea*, on 28th of March 2019, just a few weeks before the adults of the phylophagous generation of *P. oleae* were expected to appear, two different traps to capture and to monitor both adult populations of *P. oleae* and *C. carnea* at each of the sampling points were placed. To collect the olive moth, one funnel trap was located at each sampling plot. It had a closed pot shape, having a support at the top to place a specific sexual pheromone to attract the olive moth (Z-7-tetradecenal) which was replaced every 6 weeks. Inside, we poured approximately 150 mL of glyco-ethylene to retain and preserve captured insects. To capture adults of the predator, one McPhail trap per plot was used. Inside, it had a liquid content consisting of a 250 mL aqueous solution with 5% diammonium phosphate and 2% borax, which is very effective in attracting *C. carnea* as well as other insects. The predator abundance considered the entire *C. carnea* complex. We acknowledge that *C. carnea* is a cryptic species that includes several species [42,43], however due to the high abundance of collected organisms an identification was not carried out at such a level, therefore the effect of each species of the *C. carnea* complex was not evaluated in this study. In both traps, the collection of the organisms was done every two weeks, however their liquid contents were replaced every 4 weeks, because during this period they still maintained their characteristics. The traps were placed in a central position of each olive grove, hanging on tree branches and separated by fifty meters which corresponds to each trap radius of action, thus avoiding influencing the predator–prey relationship.

The organism’s collection lasted until the 18 July 2019. This was the moment in which the disappearing of the anthophagous generation adults from the olive moth traps was noticed. During this period (28 March–18 July), they have laid the eggs of the carpophagous generation that causes serious damage to the olive production and to the future development of the olive tree. Also, during this period, those eggs were susceptible to be preyed by *C. carnea*. Knowing the pest and predator abundance values allows the understanding of the dynamics of the predator populations in the olive groves during the periods of greatest activity of the pest. The captured insects were stored in flasks with 70% alcohol, duly identified, and then examined in the laboratory, where the different populations of captured insects were screened and the number of individuals of both *P. oleae* and *C. carnea* populations were counted.

To determine pest infestation levels of *P. oleae*, twenty olive fruits were collected per tree, homogeneously captured around the tree canopy. The olives were collected after a random selection of ten olive trees per olive grove, excluding the two olive trees that contained the two traps. A total of two hundred olives per olive grove were properly bagged, identified and later analyzed in the laboratory. The olive fruits were collected on 20 June 2019 when the adults of the anthophagous generation population started to rise because at that point they were laying eggs, allowing us to see the infestation at that moment. This is a well-known method that is applied to monitor pest population and to make decisions about when to apply insecticide under IPM programs. It is also used to estimate potential harvest losses [28]. Through the attack of the olive moth, it was possible to record the parameter level of infestation, corresponding to the sum of olive fruits containing eggs per olive grove.

### 2.3. Statistical Analyses

To perform the analysis two different datasets were generated, one containing the abundance values of *C. carnea* and *P. oleae* recorded over the trial period (25 olive groves by 9 recording periods giving a total of 225 observations), and another one where we aggregated values of abundance by averaging them by olive grove. These aggregated data were merged with the data from the fruit infestation by the olive moth antophagous generation egg laying as we only had one sampling date for this value of infestation.

An initial exploration of data was done through exploratory work on distributions and correlations, setting the collinearity criteria at a level of r = 0.5. After carrying out exploratory work, the factors that have the most potential to be used as reference variables were, besides *C. carnea* and *P. oleae* abundances, the Shannon diversity index, and the percentage of oak forests, pine forests, eucalyptus plantations, grasslands, shrublands and vineyards (Figure S1). However, a high correlation between surrounding olive groves and Shannon Diversity index (r = −0.77) was found so we decided not to include those two variables in the same statistical models to avoid collinearity problems.

To achieve the objective presented in this work, different data analysis approaches were used. First, to account for the factors that determine the presence of *C. carnea* adults in olive groves, an inferential generalized additive mixed model (GAMM; package “mgcv”) [44] was created in which the abundance of *C. carnea* adults was included as response variable and as predictor variables the time in Julian days, the abundance of adults of *P. oleae*, the percentage of surrounding land-uses (oak forests, pine forests, eucalyptus plantations, grasslands, shrublands and vineyards) and the Shannon’s diversity index per olive grove. A Poisson error distribution was used as our response variable is a count. Finally, as different samples were collected in the same location, the olive grove identity was used as a random factor. To perform this model, the non-aggregated dataset was used.

A similar approach was used for the abundance of *P. oleae* in which the olive moth abundance was included as response variable. The complete sampling period in Julian days, the different percentages of surrounding land-uses as well as the Shannon’s diversity index by olive grove were predictor variables. However, in this model, *C. carnea* abundance was not included as predictor, due to the notice of a reverse causality effect. As we mentioned before, the surrounding olive groves variable was very correlated with the Shannon’s diversity index variable so was not advisable to include it in this model. However, as this work also wants to study the resource concentration hypothesis, surrounding olive groves should be included in order to test if a concentration of optimal resources for the pest (i.e., olive groves surrounding olive groves) would have an effect on it. For that reason, we decided to create another generalized additive mixed model in which the abundance of *P. oleae* was included as response variable and as predictor variables were only included the complete sampling period in Julian days and the percentage of olive groves surrounding the sampling point by olive grove. Similar to the *C. carnea* model, a Poisson error distribution was used as the error distribution for both olive moth abundance models. To account for heteroskedasticity we plotted the residuals versus the fitted values finding no pattern [45]. We also checked for overdispersion by testing it with the (package “AER”) [46] finding no over or under-dispersion.

To account for the effect of *C. carnea* abundance and percentage of surrounding land-uses on *P. oleae* infestation we opted for a model selection approach through Generalized Linear Models (GLM; package “lme4”) [47]. We chose this approach because the number of observations of the aggregated dataset was not enough to perform the former models that contains more predictors that these ones. Such models were performed using only a portion of the data, creating a time lag with records between the day the study started (Julian day 88) and the day when the peak of the *P. oleae* population was reached (Julian day 154). This was done because the effect of the abundance of *C. carnea* on the infestation rate of *P.oleae* can account from the date when infestation data was collected. Along with the abundance of *C. carnea*, each one of the land-uses were separately included as an addition and as an interaction. Each land-use proportion alone, the abundance of *C. carnea* alone and a null model were also included in the model set, thus resulting in 26 models. To select the best model out of the 26 candidates, the Akaike Information Criteria corrected for small sample size (AICc) was used. This parameter estimates the quality of each model relative to each of the other models under comparison. The model selected for further consideration is the one with the lowest AICc of all the models proposed with a difference of two units for the next one. As the number of collected olive fruits varied, we opted for a response variable as proportion of counts with a binomial error distribution. We also checked the best model for heteroskedasticity using the same method mentioned above finding no problem with the procedure [45]. All statistical treatment of the data was performed using R programming language for statistical computing through RStudio software (RStudio, Inc., Boston, MA, USA) which operates the R language.

## 3. Results

During the entire experimental period, between Julian days 88 and 199, a total of 1004 individuals of *C. carnea* and 1394 individuals of *P. oleae* were captured. The presence of the olive moth in the olive groves was, therefore, 38.84% higher than the abundance of its predator.

### 3.1. The Effect of Different Land-Uses on P. oleae Population

**Figure 2:**
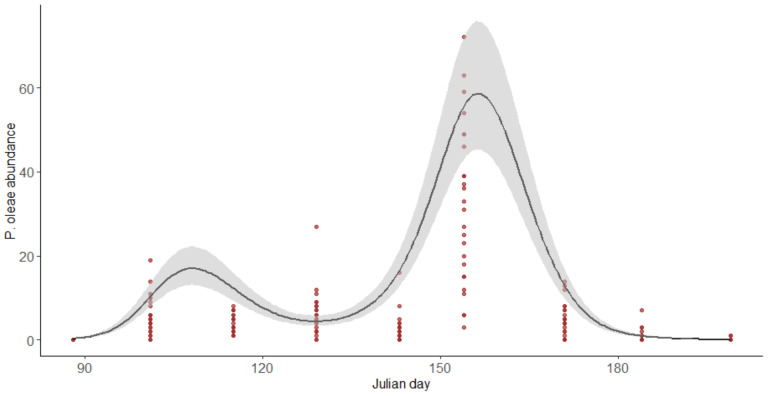
Abundance of *P. oleae* along time (in Julian days). Black solid line represents the predictions of the model for the abundance of *P. oleae*. Shaded areas represent the interval of confident at 95%.

*P. oleae* has an initial peak registered between the Julian days 101 and 115, corresponding to the phylophagous generation. This was followed by a drastic decrease on registered individuals due to the transition from the phylophagous to the antophagous generations of the pest, detected between the collection of Julian days 129 and 143. Its second and most representative peak at Julian day 154, corresponding to the antophagous generation (Figure 2).

**Figure 3:**
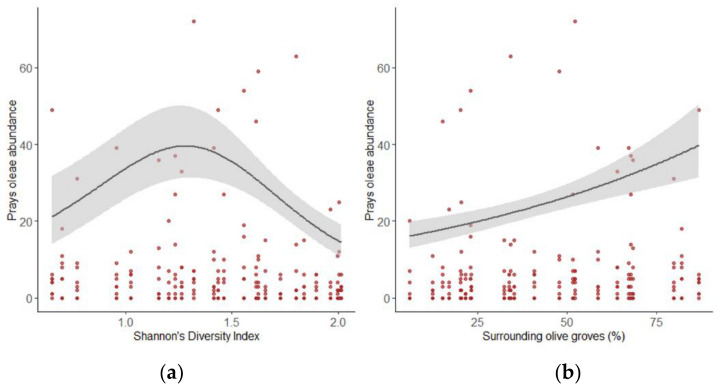
(**a**) Effects of surrounding diversity measured through Shannon’s diversity index on *P. oleae* abundance. Black solid line represents the abundance of *P. oleae*. Shaded areas represent the interval of confident at 95%; (**b**) Effects of surrounding olive groves percentages on *P. oleae* abundance. Black solid line represents the abundance of *P. oleae*. Shaded areas represent the interval of confident at 95%.

From the Generalized Additive Mixed Models, the presence of *P. oleae* in olive groves was significantly affected by Shannon’s diversity index (*p* = 0.022). Only high values of this landscape metric can decrease the abundance of *P. oleae* in olive groves (>1.2). Lower values induce an increase of olive moth values of abundance (Figure 3a).

In the same line, the abundance of *P. oleae* increases for higher percentages of surrounding olive groves (*p* = 0.022; Figure 3b).

### 3.2. The Effect of Different Land-Uses and P. oleae Abundance on C. carnea Abundance

**Figure 4:**
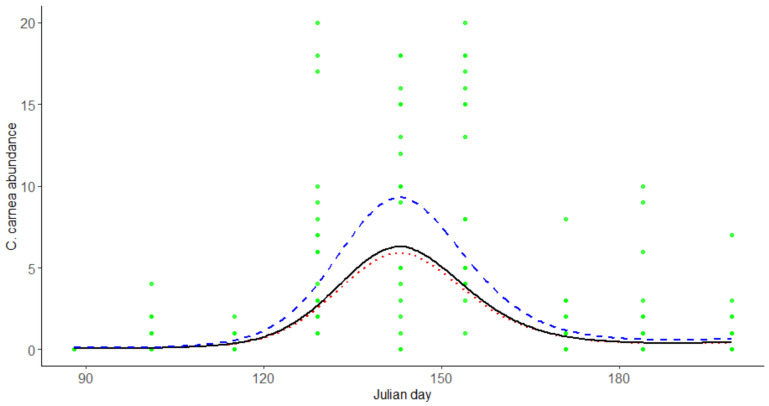
Abundance of *C. carnea* along time (in Julian days) under the influence of different *P. oleae* abundance levels. Black solid line represents the estimated abundance of *C. carnea* under the influence of *P. oleae* abundance of a quantile 50% of the observed data (2.0 individuals per trap per fifteen days). Red dotted line represents the estimated abundance of *C. carnea* under the influence of *P. oleae* abundance of a quantile 10% of the observed data (0.1 individuals per trap per fifteen days). Blue dashed line represents the estimated abundance of *C. carnea* under the influence of *P. oleae* abundance of a quantile 90% of the observed data (14.6 individuals per trap per fifteen days).

*C. carnea* abundance registered its peak at Julian day 143. *P. oleae* abundance (*p* < 0.001) was the only factor that significantly affected the presence of adults of *C. carnea* in olive groves. *C. carnea* abundance almost doubled its abundance when there was a higher abundance of the pest. However, when the abundance of the pest was medium or low, the abundance of adults of *C. carnea* was almost the same (Figure 4). In contrast, no land-use reported any effect on *C. carnea* populations.

### 3.3. The Effect of Landscape Metrics on the Level of Infestation in Olive Groves

The interaction among the percentage of surrounding olive groves and the abundance of *C. carnea* (AICc = 449.70) was the model that better explained *P. oleae* infestation (Table 1).

**Figure 5:**
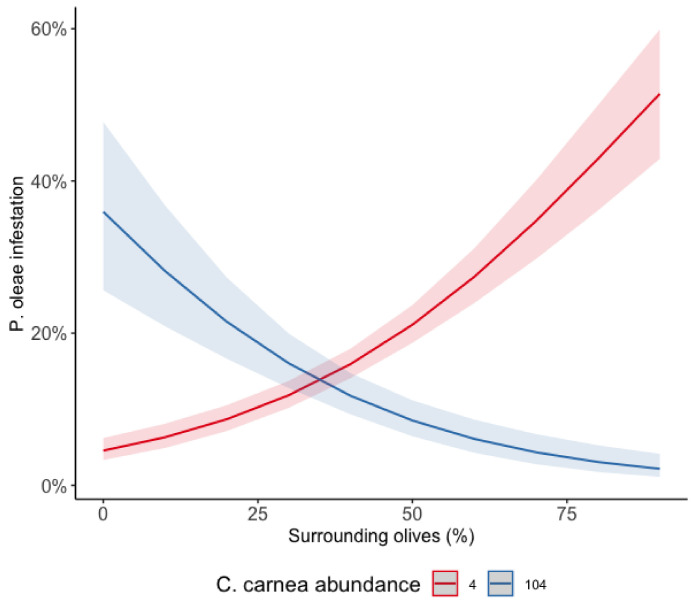
Infestation of *P. oleae* along a gradient of surrounding olive groves and different levels of *C. carnea* abundance. Red line represents the estimated effect of surrounding olive groves under a low abundance (red line) and high abundance (blue line) of *C. carnea.* Shaded areas represent the interval of confidence at 95%.

When the abundance of *C. carnea* was low, the infestation of *P. oleae* tended to increase along a gradient of surrounding olive groves (Table S1). However, when the abundance of *C. carnea* was high, the infestation of the pest tended to notably decrease from a level of around 20% to a level of 10% (Figure 5).

## 4. Discussion

In our study, we found that the abundance of the adult predator *C. carnea* increases with the greater presence of the pest *P. oleae* in olive groves, hitting its peak just a few days before the highest peak of *P. oleae*, which may indicate that the predator is directly attracted by the presence of this pest, which is an assumption in line with what has already been described [40,48]. *C. carnea* larvae are major oophagous predators known to play a predominant role in the predation of eggs layed by the antophagous generation of the olive moth [31,33]. However, the percentage of different land-uses surrounding the sampling points does not seem to have an effect on *C. carnea* population and the predator levels of abundance are more related to the olive moth presence than to the percentage of surrounding land-uses. Highly mobile organisms such as *C. carnea* are affected by landscape composition [37,38] but its dispersal abilities may also be affected by meteorological events. For example, Duelli [49] registered dispersal variations based on diel wind speed measurements. Since variation in meteorological parameters can influence *C. carnea* ability to disperse, our study would benefit from analysis to annual climatic and meteorological variations to better understand the fact of *C. carnea* population not being affected by the percentage of different land-uses surrounding the sampling points. Agricultural management practices, competition between natural enemies, and their preference for different food resources other than *P. oleae* can also be a set of factors that explain the abundance of *C. carnea* being more directly related to the presence of *P. oleae* inside the olive crops than to the different land-uses.

*P. oleae* infestation and abundance increased with the percentage of olive groves surrounding the sampling points which can be explained by the resource concentration hypothesis. This hypothesis states that expansive monocultures allow specialist pest populations to rapidly build and disperse, whereas diverse landscapes mitigate population growth and spread [14,22,24]. However, the effect for *P. oleae* infestation was counteracted when there was a high abundance of *C. carnea* in the olive groves. This is even more explicit when the abundance of *C. carnea* was low as in these situations the infestation levels of *P. oleae* continued to increase when more olive groves were surrounding the sampling points. This conclusion is also founded in the fact that olive groves with a higher abundance of *P. oleae* were those in which *C. carnea* showed higher abundances.

Hereupon, it is possible to realize that olive groves, as a land-use, have both the potential to increase the abundance of *P. oleae* and, on the contrary, when associated with high values of *C. carnea,* they have the potential to decrease infestation and, consequently, to decrease the abundance of the following generations of the olive moth. The effects of agricultural management practices such as the application of pesticides or land ploughing are known to have a negative effect on the abundance of natural enemies of pests within agroecosystems [2,50]. During this study, agricultural managers did not apply pesticides and did not use land ploughing methods in any of the twenty-five olive groves where sampling points were located. Therefore, as there was no interference from these factors, it explains the predictable significant effect of *P. oleae* on the attraction of its natural enemy, *C. carnea*. These findings allow us to confirm the biological control potential of *C. carnea* as it can control the infestation of this pest as well as it feels attraction for it [10,31,32,33,40].

Using class-level landscape metrics such as Shannon’s diversity index, it was observed that greater diversity in the vicinities of the crop tends to decrease the abundance of *P. oleae*. The most conceivable explanation is based on the natural enemy hypothesis, which recognizes that many natural enemies of crop pests (i.e., predators and parasitoids) depend on a diversity of crops and/or natural habitats for alternative food resources, overwintering, etc. Thus, more diverse landscapes may facilitate better pest control [8,51,52]. In the same line, Villa et al., (2020) [53] related the landscape diversity and configuration at larger scales with a decrease of *P. oleae* abundance. Some authors have suggested that natural or semi-natural habitats at the landscape scale are important elements that favor the prospection of natural enemies of olive pests by providing undisturbed areas that offer shelter from crop disturbances as well as overwintering refuges, alternative hosts, and prey, and additional food resources [7,19,54]. The assemblage of natural enemies of *P. oleae* not only includes *C. carnea,* but also other common predators of the olive moth such as ants, Coleoptera, Hemiptera, and spiders [55]. As described by Paredes et al. (2015) [56], effective assemblages of natural enemies are better suppressing a Lepidopteran pest, such as *P. oleae* than a species of natural enemies acting alone. For a Lepidopteran pest with a complex life cycle, the single best predator taxon was markedly poorer at suppression than the most effective assemblage. As an example, *Anthocoris nemoralis* biological control effectiveness on *P. oleae* was strongly related with its abundance being positively influenced by natural habitat [57].

## 5. Conclusions

This study identifies chain relationships that confirm the attraction of the predator, *C. carnea*, to the olive moth, *P. oleae*. It suggests the biological control potential of *C. carnea* in olive groves and identifies its potential to reduce harmful effects of *P. oleae* in olive groves. While olive groves themselves have a direct contribution to the increase in the abundance of olive moth, our study highlights that promoting landscape diversity through increasing diversity of land-uses in the vicinities of olive groves directly affects the abundance of this pest by decreasing it. Although in-depth knowledge is needed about which plants and semi-natural habitats are the best ecological infrastructures to increase the proliferation of natural enemies and avoid further pest pressure, the prospects are that the enhancement of land-uses diversity can help olive growers to improve and make their production healthier by doing their integrated pest management through conservation biological control strategies.

The conceptions originated from this study seek to rethink the formation of agroecosystems and are intended not only to complement the existing literature on conservation biological control methods but also to create a robust knowledge foundation that provides both olive growers and policy makers with relevant information that they can apply in order to improve and assign greater value to their production economy and to meet the increasingly demanding and necessary environmental standards through alternatives to the use of pesticides without causing damage to the environment, instead promoting public health.

## Figures and Tables

**Table 1:** AICc models comparison for the studied variables of the *P. oleae* carpophagous generation eggs infestation on olive fruits during the complete study period and until the time lag settled at the 154 Julian day. The model with the lowest value of AICc is considered as the best model and conclusions are based on it. In bold best model AIC.

Model Type	AICc
Null Model	554.02
*C. carnea*	550.58
Eucalyptus plantations	549.72
Eucalyptus plantations + *C. carnea*	549.82
Eucalyptus plantations × *C. carnea*	538.85
Grasslands	542.97
Grasslands + *C. carnea*	532.63
Grasslands × *C. carnea*	500.81
Oak forests	555.71
Oak forests + *C. carnea*	552.59
Oak forests × *C. carnea*	551.89
Shrublands	552.34
Shrublands + *C. carnea*	546.73
Shrublands × *C. carnea*	548.72
Vineyards	513.40
Vineyards + *C. carnea*	514.09
Vineyards × *C. carnea*	506.92
Olive Groves	537.42
Olive Groves + *C. carnea*	530.84
Olive Groves × *C. carnea*	**449.71**
Pine Forests	545.28
Pine Forests + *C. carnea*	538.09
Pine Forests × *C. carnea*	538.72
Shannon’s diversity index	543.25
Shannon’s diversity index + *C. carnea*	536.85
Shannon’s diversity index × *C. carnea*	505.53

## Supplementary Materials

The following are available online at https://www.mdpi.com/2075-4450/12/1/46/s1, Figure S1: Correlations of the different predictor variables included in the models. Table S1: Estimated parameters of the selected model for the *Prays oleae* infestation.
