# Supplementary material for: Land-Use Effect on Olive Groves Pest Prays oleae and on Its Potential Biocontrol Agent Chrysoperla carnea
Source: Insects. 2021 Jan 8;12(1):46. doi: 10.3390/insects12010046 (PMC7827753; doi:10.3390/insects12010046)
Supplement: Supplementary file 1 [file insects-12-00046-s001.pdf]

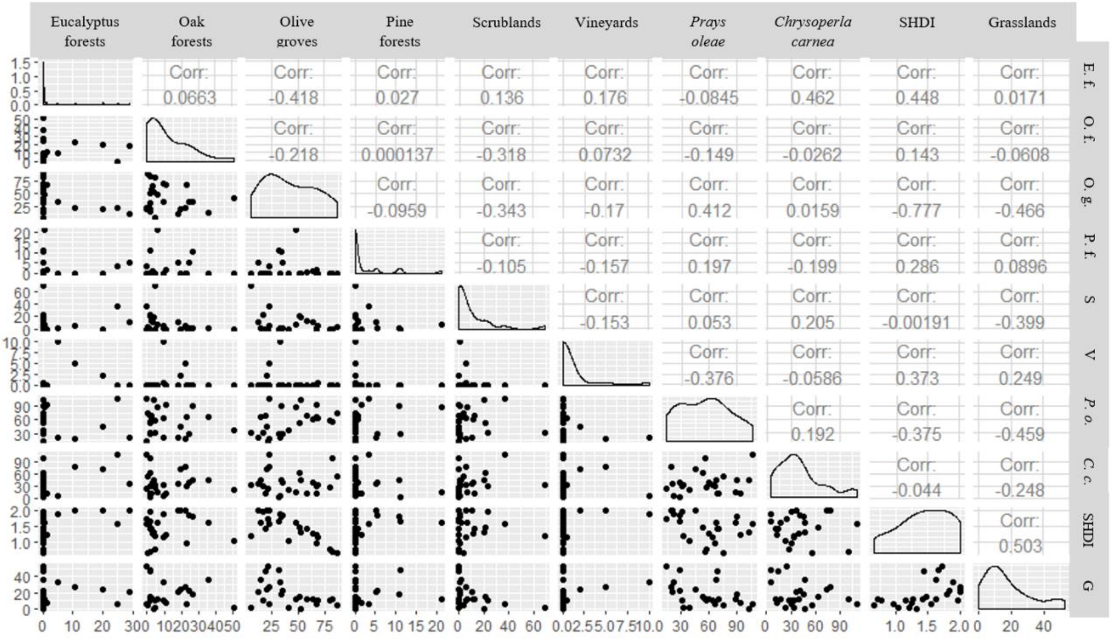

Figure S1. Correlations of the different predictor variables included in the models.

Table S1. Estimated parameters of the selected model for the *Prays oleae* infestation. Pr (<|z|) shows the level of support of these parameters.

	**Estimate**	**Pr (<|z|)**
Intercept	−3.1380	<0.001
Surrounding olive groves (%)	0.0372	<0.001
C. carnea abundance	0.0246	<0.001
Interaction (Olive groves × *C. carnea*)	−0.0007	<0.001
